# Being a Gambler during the COVID-19 Pandemic: A Study with Italian Patients and the Effects of Reduced Exposition

**DOI:** 10.3390/ijerph18020424

**Published:** 2021-01-07

**Authors:** Maria Anna Donati, Silvia Cabrini, Daniela Capitanucci, Caterina Primi, Roberta Smaniotto, Maurizio Avanzi, Eleonora Quadrelli, Giovanna Bielli, Alfredo Casini, Alessandra Roaro

**Affiliations:** 1 Department of Neuroscience, Psychology, Drug, and Child’s Health, Section of Psychology, University of Florence, 50135 Florence, Italy; caterina.primi@unifi.it; 2 National Health Drugs Service (Servizio Dipendenze Patologiche, Ser.DP) of Piacenza, Azienda Unità Sanitaria Locale AUSL Piacenza, 29121 Piacenza, Italy; silvia.cabrini1@gmail.com (S.C.); maurizioava@hotmail.com (M.A.); 3 Association AND-Azzardo e Nuove Dipendenze [Gambling and New Addictions], 21013 Gallarate, Italy; capitanucci.daniela@gmail.com (D.C.); smaniottoroberta@gmail.com (R.S.); 4 Cometa Consorzio di Cooperative Sociali [Consortium of Social Cooperatives], 19121 La Spezia, Italy; ele0503@hotmail.com; 5 National Health Drugs Service (Servizio Dipendenze Ser.D.) of Parabiago (Milan), Azienda Socio Sanitaria Territoriale, ASST Ovest Milanese, 20025 Legnano, Italy; gbielli@libero.it (G.B.); alessandra.roaro@gmail.com (A.R.); 6 National Health Drugs Service, Mental Health and Addiction Department (SERT) of La Spezia, ASL Azienda Sociosanitaria Ligure 5, 19121 La Spezia, Italy; alfredo.casini@asl5.liguria.it

**Keywords:** COVID-19 pandemic, gambling disorder, environmental prevention, gambling restrictions, problem gamblers, harm reduction, abstinence, telephone interview, treatment, Italy

## Abstract

The COVID-19 pandemic, with the consequent lockdown of about 3 months, can be viewed as an experimental model to observe the impact of the depletion of environmental factors that stimulate gambling, particularly electronic gambling machines (EGMs) that were set to zero. The effects of some structural characteristics of gambling activities that increase gambling behavior were studied among disordered gamblers in treatment in this unique scenario. In fact, studies investigating the effects of the lockdown on problem gamblers (PGs) under treatment are missing. The aims of this study were to analyze patients’ gambling behavior and craving during the lockdown and to conduct a comparison between gambling disorder (GD) symptoms at the beginning of the treatment and during lockdown. The study was conducted in Italy, the European country with the largest gambling market and the first to be affected by the virus. Data were collected through a semi-structured telephone interview conducted by healthcare professionals. Participants were 135 PGs under treatment (109 males, mean age = 50.07). Results showed that most PGs achieved a significant improvement in their quality of life, with less gambling behavior, GD symptoms, and lower craving. No shift toward online gambling and very limited shift towards other potential addictive and excessive behaviors occurred. The longer the treatment, the more monitoring is present and the better the results in terms of symptoms reduction. Individual and environmental characteristics during the lockdown favored the reduction in symptoms. Consideration for prevention and treatment are discussed.

## 1. Introduction

During the past two decades, gambling availability has strongly increased as a consequence of socio-economic and political decisions highly oriented towards promoting different gambling forms. Therefore, participation and expenditure have increased, with substantial growth in the prevalence of gambling disorder (GD) and gambling-related harms [1,2,3]. The harms from gambling are wide-ranging, affecting people and their family’s resources, relationships and health. For these reasons, both clinicians and investigators are beginning to highlight it as a major global health issue [1].

Given that GD is a multifaceted disease, the study of its determinants and the search for vulnerability factors have been developed in three main areas: the individual risk factors; the structural characteristics of gambling devices; and the ‘gamblogenic’ harmful environments [4]. Among the risk factors, the individual component is unquestionably the most studied. Dowling and colleagues [5] systematically reviewed risk and protective factors for problem gambling, highlighting several individual risk factors such as male gender, low school performance, antisocial behaviors, depression, impulsivity, substance and alcohol use, number of gambling activities, problem gambling severity, sensation seeking and violence. As for the gambling products, for instance, the structural characteristics of electronic gambling machines (EGMs) are now known to incorporate features that increase reinforcement or stimulus and thus engender increased use and addiction [6]. Finally, monitoring of the environment is important because it appears that there is a positive relationship between access to gambling sites and the adoption of gambling habits in neighboring communities [4,7]. However, we have insufficient knowledge about what occurs when the gambling-inciting environment cannot exercise its influence on individuals’ gambling behavior.

In this regard, the global pandemic due to the coronavirus disease 19 (COVID-19), officially declared as such on 11 March 2020 by the World Health Organization (WHO, Geneva, Switzerland), with the consequent lockdown provides an experimental model with which to observe the impact of the depletion of environmental factors that stimulate gambling, in particular due to the unavailability of most land-based gambling during the lockdown.

Contemporary research has attested that the outbreak caused by COVID-19 resulted in serious health and psychosocial problems worldwide, manifesting as psychological distress associated with duration of lockdown, fear of infection, feelings of frustration and boredom, inadequate information and inadequate supplies [8]. The persistent lack of social interaction has also proved to be seriously disturbing for human beings [9]. Wenjun Cao and colleagues [9] have found that during the epidemic, students reported increased anxiety, positively moderated by economic stability and reduced isolation (i.e., living with parents).

However, the specific effects of the lockdown on gambling behavior have been poorly analyzed. Some countries such as Italy, England, Canada and Australia had most gambling venues closed after the outbreak and most countries have reduced the opportunity of access. Moreover, as a corollary to the containment measures, given that online gambling offers remained unchanged or even increased, a shift to online platforms was observed [10]. As a stress-based approach has shown that both anxiety and boredom are associated with problem gambling [11], both of which can be expected to increase during uncertain and threatening times [12], some researchers have postulated an increase in addictive behaviors and a switch to online gambling [13]. Instead, an environmental approach would suggest that a decrease in availability could calm down the need for gambling [14,15]. In England, the UK Gambling Commission [16] observed that a few players who were already engaged in gambling diversified their gambling activities, gambled longer per session or spent more money on gambling. Nevertheless, fewer consumers were gambling and the incidence of switching to online gambling was relatively low [17]. In Australia, the general population has been surveyed to monitor gambling behaviors. The majority of participants reported gambling less frequently during the shutdown and not increasing their online gambling frequency. They also reported that psychological distress and COVID-related financial difficulties led to increases in gambling expenditure but not in gambling frequency [15].

In Italy, the gambling environment is notable [18]; to be more precise, it has become the largest gambling market in Europe and the fifth largest in the world after the United States, Japan, China and Macau [19]. These five countries account for 54% of world gambling revenues, with Italy accounting for 5% of the global revenues [20]. As a consequence, the number of problem gamblers in Italy is high—3% of the global population [21]. Moreover, Italy, among Western countries, had an early diffusion of the COVID-19 virus, and drastic social containment measures were implemented quickly. In fact, the lockdown has entailed a complete closure of the majority of land-based gambling venues for nearly two months, with only the exclusion of scratch cards. Nevertheless, there are a few Italian studies on gambling during the lockdown period, and they were threefold, focusing on the general population, on problem gamblers (PGs) not seeking treatment and PGs in treatment. There was a significant reduction in the total amount of money spent on gambling compared to the previous half-year period (30% lost). Specifically, the reduction has been mainly due to a loss in land-based gambling devices (55% lost), whereas online gambling has had an increase in money spent (25.4%) [22], even though the cancellation and postponement of sports events in Europe has reduced sports betting [23]. In April–May 2020, a survey on the general population monitored the consequences of the COVID-19 outbreak and the spread of gambling behavior. An increase in frequency and expenditure for online gamblers was observed but no shift from land-based to online gambling was reported [24]. The Italian National Health Service Phone Counselling Service for gambling problems has reported data on non-treatment-seeking PGs calling for support. Gamblers reported feeling distressed at not being able to gamble during the lockdown, experiencing problems concerning past debts and worrying about new debts caused by the pandemic [25]. With regard to PGs in treatment, two regions in Italy surveyed clients in treatment for PG in their health services. During the lockdown period, requests for treatment for PG greatly decreased, whereas there was a slight growth in people asking for economic counselling. Clients already in treatment for PG had a positive attitude about restrictions on gambling venues, and this had positive consequences on their personal and familial environments. They reported no switch from land-based to online gambling [26,27].

Following these premises, the general goal of this work was to investigate the psychological and behavioral effects of the COVID-19 lockdown on Italian pathological gamblers under treatment in order to gain information regarding gambling prevention and treatment. Given the impossibility of treating patients during quarantine, it has been recommended to preserve a clinical connection to mitigate the possible effects of the lockdown [28], and several medical sectors have tried to implement the idea of taking remote clinical action [29]. Thus, we developed a semi-structured telephone interview to be conducted by the healthcare professional in charge of the patients before the lockdown as a monitoring tool to orient supporting actions.

In detail, the aims of our study were threefold. First, we aimed to describe patients’ life conditions during the lockdown, with particular attention to patients’ gambling behavior, gambling-related emotions, GD symptoms and gambling craving. We were also interested in knowing the patients’ personal perceptions of changes in their life because of the quarantine, when nearly all land-based gambling options were absent. Based on the above-cited data, we expected a positive effect of the diminished gambling opportunity despite the difficult conditions. Second, we aimed to investigate the relationships between gambling during the lockdown, variables related to the pre-lockdown period and life conditions in the lockdown in order to understand which past and present individual and environmental factors were associated with gambling during the lockdown.

Our third aim was to explore whether the effects of the treatment were maintained despite the stressful context and whether the absence of land-based gambling opportunities was protective by conducting a comparison between GD symptoms at the beginning of the treatment and during the lockdown.

## 2. Methods and Materials

### 2.1. Participants

Participants were 135 pathological gamblers under treatment (109 males and 26 females, mean age = 50.07, SD = 13.33, range: 22–78 years) recruited in the north of Italy. The majority were contacted through the National Health Drugs Services (Ser.D.); overall, 44% (*n* = 60) were under treatment at Ser.D. of La Spezia (Liguria), 31% (*n* = 42) at Ser.D. of Piacenza (Emilia Romagna) and 18% (*n* = 25) at Ser.D. of Parabiago (Milan, Lombardy); an additional 7% (*n* = 8) were recruited at the private no-profit association AND-Azzardo e Nuove Dipendenze [Gambling and New Addictions] in Gallarate (Lombardy).

Concerning education level, 5% (*n* = 7) had stopped education at elementary school, 43% (*n* = 58) had a middle school diploma, 47% (*n* = 63) had a high school diploma and 5% (*n* = 7) had a university degree. Most patients were married (33%, *n* = 44) or single (29%, *n* = 38). Sixteen percent (*n* = 21) were cohabitant, 15% (*n* = 20) were separated/divorced and 8% (*n* = 9) were widowed. As for the familial status, the majority (54%, *n* = 72) lived with a partner (with or without sons), 26% (*n* = 34) lived with their original family, 19% (*n* = 26) lived alone and 1% (*n* = 1) lived in a residential structure. With regard to the occupational status, 10% (*n* = 14) were unemployed, 19% (*n* = 25) were retired, 1% (*n* = 2) were students and 70% (*n* = 94) were employed. We classified participants who worked based on the Italian National Institute of Statistics (ISTAT), which divides the population into nine categories. These categories are as follows: legislators, managers and entrepreneurs (*n* = 0); intellectual, scientific and highly specialized professions (2%, *n* = 2); technical professions (6%, *n* = 5); employees (20%, *n* = 17); business and service professions (17%, *n* = 15); artisans and farmers (38%, *n* = 33); plant and semi-skilled workers of fixed and mobile machinery (7%, *n* = 6); freelancers (7%, *n* = 6); armed forces (3%, *n* = 3).

Table 1 reports information of the sample in relation to treatment and gambling behavior before the beginning of the lockdown. Patients were under treatment for an average time of about seven years, and most of them were in the course of treatment or in the monitoring phase. The most frequent treatment approach was psychological treatment, and the majority of patients had experienced abstinence from gambling, but half of them had relapsed. Patients gambled, on average, on about two activities, and the most prevalent was slot machines, on which they gambled predominantly on a regular basis. All of them were classified as disordered gamblers based on *the Diagnostic and Statistical Manual of Mental Disorders—Fifth Edition* [30] criteria for GD and following the classification of gambling problem severity based on various instruments used in the different health services such as the South Oaks Gambling Screen (SOGS) [31,32], used in three of the four services, the Canadian Problem Gambling Index [33,34], employed in one service, and the Kurzfragebogen zum Glücksspielverhalten [35,36], applied in one out the four services. The participants could be mostly classified as Pathways 1 and 2 according to the Pathways Model [37,38]. About 40% of the patients had comorbidities, the most widespread of which were depressive disorders and personality disorders, following the DSM-5 disorders classification.

### 2.2. Procedure

To collect data during the lockdown, a semi-structured telephone interview to be conducted by the healthcare professionals with the patients was developed. In each recruitment center, a list of eligible patients to be contacted was prepared, and the interview was then realized by the healthcare professionals who were treating the patients before the beginning of the lockdown. Two patients were contacted but did not offer their consent to undergoing the interview. The patients who participated in the study completed the interview anonymously and only after having understood the information sheet and having given their informed consent. The interviews lasted about 40 min and were conducted throughout the Italian lockdown phase, specifically from 7 April 2020 to 28 May 2020.

### 2.3. The Telephone Interview

The telephone interview was articulated in five sections (see Supplementary Materials Table S1 for a detailed description of the interview questions). The first section was focused on the patients’ life conditions during the lockdown (home environment, familial relationships and money management), emotional state during the lockdown and behavioral intentions for the post-quarantine period.

The second section regarded the personal relationship between the patients and the COVID-19 pandemic. The perceived emotional impact of the COVID-19 pandemic was also measured. To assess the patients’ adherence to the national restrictions, their attitude and behavior were investigated. To assess whether the patients’ behavior adhered to the rules, we investigated the frequency of exits from home in the last week and reasons for the exits.

The third section investigated gambling behavior with related pathological symptoms, gambling-related emotional states and gambling craving. Gambling frequency and problem gambling were assessed through the SOGS [31,32] as it was the most widely used measurement instrument for gambling problem severity classification across the four health services involved in the study, which is consistent with what happens in the National Health Drugs Services in Italy [36]. The SOGS is a 20-item questionnaire based on the DSM *Third Edition* [39] criteria for problem gambling. It is a widely used screening instrument for problem gambling and shows good reliability and validity in community and clinical samples [40,41]. For the study purposes, we omitted the non-scored items 2 and 3, investigating the largest amount of money ever gambled with on any one day and parents’ gambling problems, respectively. We also modified the original time frame by referring to the last month. The first item of the SOGS investigated the frequency of gambling (not at all = 0, less than once a week = 1, once a week or more = 2) in ten activities, including playing cards for money, betting on horses, dogs or other animals, sport bets, dice games for money, casino, betting on traditional/instant lotteries, bingo, stock and/or commodities market bets, slot machines, poker machines or other gambling machines and games of skill for money. To obtain further information about gambling frequency, also by taking into account the specificity of the lockdown period in the closure of legalized gambling venues, we also included online games for money and private bets with friends and family members. Based on their responses to those items, participants were classified as non-gamblers (no gambling behavior) or gamblers (gambling on at least one activity; [42]). Among gamblers, non-regular gamblers (i.e., those who participated less than once a week in at least one gambling activity) and regular gamblers (i.e., those who participated once a week or more in at least one gambling activity) were identified [43]. A total score of gambling frequency (range: 0–24) was obtained by summing the responses for each activity. To have a measure of problem gambling, the responses to the items investigating gambling problem symptoms were summed. An example of the items is: “Have people criticized your gambling?” Each of those items required a dichotomous answer (i.e., yes = 1 or no = 0), except three items, which have a 4-point or 3-point response scale dichotomized (i.e., never = 0, some of the time (less than half the time)/most of the item I lost/every time I lost = 1; never (or never gamble) = 0, yes, less than half the time I lost/yes, most of the time = 1; no = 0, yes, in the past, but not now, yes = 1) in the scoring phase. A cut-off score of 5 or more indicates that the respondent is a PG [31].

Emotional states towards gambling and gambling craving were also investigated.

The fourth section focused on the frequency during the last month of eight potential addictive behaviors. We took into account not only behaviors that, if excessively practiced, can lead to disorders as defined in the Substance-Related and Addictive Disorders section of the DSM-5, including alcohol, tobacco and substance use, but also other behaviors that can develop into problematic use. In particular, we also assessed the frequency of videogame playing [44], Internet use [45,46,47], smartphone use [48], online shopping [49,50] and TV watching [51].

The final section of the interview was aimed at relieving the patients’ perceptions about changes from the pre-lockdown period to the ongoing lockdown period concerning various aspects: relationships with family members, general emotional state, money management under the supervision of family and frequency of other potential addictive behaviors.

## 3. Results

### 3.1. Description of the Gamblers during the Lockdown

#### 3.1.1. Life Conditions

During the lockdown, participants lived in medium-size houses (M = 99.29, SD = 45.08, range: 40–300 m²). Ninety-two percent of the patients (*n* = 121) had houses with open spaces: terrace(s): 58% (*n* = 78); garden(s): 58% (*n* = 77); vegetable garden(s): 16% (*n* = 21). Twenty-two percent (*n* = 29) lived alone in the lockdown, 30% (*n* = 40) lived with another person, 30% (*n* = 40) lived with two other persons and 18% (*n* = 26) lived with more than three persons (M = 1.57, SD = 1.20, range: 0–5 persons). The relationships with the cohabitants were rated as quite good (M = 7.73, SD = 1.82, range: 1–10).

With regard to work, 70% of the patients who were employed still worked during the lockdown. Among them, 82% (*n* = 53) worked on-site and 18% (*n* = 12) were in smart working. Most of the patients (97%, *n* = 131) made use of money during the lockdown; among them, the majority used money to do the shopping (84%, *n* = 101), and the others to buy cigarettes, something at the pharmacy, to get petrol and to recharge their mobile phone, with one patient who declared using money to buy instant scratch-cards.

As for the general emotional state during the lockdown, participants’ responses were classified in different categories, based on the reported words. Fifty percent (*n* = 66) of the responses reflected a general positive state, as evidenced by the terms “*well*, *better*, *calm*, *serene*, *happy*”, while 24% (*n* = 32) of patients reported a general negative state: “*tired*, *bored*, *depressed*, *sad*, *thoughtful*”. Three percent (*n* = 4) specified being reflective in the lockdown, 9% (*n* = 12) particularly emphasized being in a state of anger regarding freedom restrictions as well as having anxiety about their work conditions due to the lockdown, 8% (*n* = 9) reported having mood swings and 5% (*n* = 9) did not specify. Two patients (1%) made an explicit reference to gambling, one reporting sadness for his past gambling behavior and the other declaring that they were keeping better because of it being impossible to gamble. With regard to behavioral intentions for post-quarantine, the most prevalent desire was to see friends (27%, *n* = 34), followed by doing sport activities/hobbies, walking (17%, *n* = 21), going on holidays (14%, *n* = 18) and returning to work (14%, *n* = 18). There were also some who intended to maintain the changes in gambling behavior verified during the lockdown after the quarantine (11%, *n* = 14). Eleven patients (9%) wanted to do shopping/services, nine (7%) intended to go to a bar and only one (1%) a poker room.

#### 3.1.2. Personal Relationship with the COVID-19 Disease and Related Restrictions

None of the sample participations were sick due to COVID-19, and almost none of the patients went into preventive quarantine because of contact tracing. However, about 40% of the patients knew someone—on average, three persons—who was affected by the virus and 10% even knew someone who had died from the COVID-19 disease—on average, three persons. Fear, stress and anxiety were the strongest emotions elicited by the pandemic. However, attitude towards the government’s restrictions was generally favorable as the average score was higher than the theoretical mean of the scale. During the previous week, 96% of the participants had gone outside the home, mostly to do the shopping, to go to work or to go to the pharmacy—thus, to do permitted actions. Everyone had, on average, almost two reasons to exit from home (Table 2).

#### 3.1.3. Gambling Behavior, Gambling-Related Emotions and Gambling Craving

Only 6% (*n* = 8) of the patients had gambled: six on traditional/instant lotteries, one on stock and/or commodities market bets and one on online games. Only one patient gambled on a regular basis, on traditional/instant lotteries. The average score relative to gambling frequency was very low (M = 0.07, SD = 0.28, range: 0–2).

With regard to gambling problem severity, the mean score at the SOGS was very low (*n* = 127, M = 1.31, SD = 1.63, range: 0–10). In particular, 16% (*n* = 20) exhibited no symptoms, 69% (*n* = 87) showed one symptom, 7% (*n* = 9) two symptoms, 3% (*n* = 4) three symptoms, 2% (*n* = 2) four symptoms, 1% (*n* = 1) six symptoms, 2% (*n* = 2) eight symptoms and 2% (*n* = 2) ten symptoms. Thus, only five patients (4%) showed problem gambling behaviors during the lockdown. Table 3 shows the percentage of endorsement by symptoms. Although the symptoms had a generally low endorsement, the feeling of having a gambling problem persisted in the patients, as well as feeling guilty because of gambling, even if this had a very lower percentage of endorsement.

Responses to the open questions investigating emotional states towards gambling during the lockdown were classified in different groups, as reported in Table 4. There appeared to be a general indifference and loss of interest towards gambling and a wide spread of oppositive emotional states. Only three patients protested against the closing of gambling venues, were bored without gambling or had ambivalent emotions.

Gambling craving resulted in being low, as the mean score of the scale (M = 11.23, SD = 6.37, range: 8–40) was very much lower than the theoretical mean value of 36.

#### 3.1.4. Frequency in Other Potential Addictive Behaviors

During the previous month, patients were involved in other potential addictive behaviors. Indeed, 94% of them watched TV (30% sometimes, 64% often), 88% stayed on their mobile phone (38% sometimes, 50% often), 76% drank alcohol (50% sometimes, 26% often), 72% stayed on the Internet (36% sometimes, 36% often), 65% smoked (12% sometimes, 53% often), 33% played videogames (22% sometimes, 11% often), 24% did online shopping (23% sometimes, 1% often) and 8% used substances (6% sometimes, 2% often).

#### 3.1.5. Perceived Changes from the Pre-Lockdown Period to the Ongoing Lockdown

As for the relationships with family members, 56% (*n* = 62) of the patients thought that their relationships were improved, 11% (*n* = 12) thought that they were worsened and 33% (*n* = 37) thought that they remained the same as before the lockdown. The patients who viewed their familial relationships as improved justified these ratings as being due to more closeness, contact, union and time spent on being and doing things together, better communication, more collaboration, more kindness, better mood, fewer fights and less job-related stress. Some of them related the improved relationships with their gambling interruption due to the lockdown, as indicated by these comments: “Since I don’t gamble anymore, I’m more lucid”, “As I do not gamble”, “I don’t gamble and therefore I have better relationships”, “Because of slot-machines closure”, “Greater freedom due to the gambling venues closure”, “I behave better without gambling”, “I don’t gamble, so I don’t tell lies”, and “No tension due to gambling”. Instead, the patients who viewed their familial relationships as worsened justified these ratings due to stress resulting from the restrictions, tensions, a desire to be alone, relational problems/misunderstandings, economic problems and a need for own space.

With regard to the perceived comparison about the general emotional state, 46% (*n* = 62) of the patients reported feeling better than, 25% (*n* = 34) worse than and 29% (*n* = 39) the same as before the lockdown. The patients who viewed their general emotional state as improved justified these ratings as being due to feelings of relaxation, tranquility and self-efficacy, economic savings, better familiar relationships, less work-related stress and also new hobbies and interests and greater reflectivity. Some also referred to gambling in terms of having goals and thoughts other than gambling, as well as the inability to gamble. The patients who rated their general emotional state as worsened justified these ratings as being due to preoccupation toward the work and economic crisis, lack of friends/family, boredom, mourning, depression, loneliness, not being able to go out—especially to the bar—anxiety and anger.

Regarding money management under the supervision of family among those who used money (*n* = 128), in comparison to the period before the beginning of the lockdown, the majority (84%, *n* = 107) did not observe changes, 9% (*n* = 12) reported that they used money less than before and 7% (*n* = 9) used more than before.

In terms of the perceived changes in the frequency of other potential addictive behaviors, watching TV resulted in being the behavior which a higher percentage of participants who declared doing it more than before the lockdown (53%, *n* = 72) compared to the percentage of participants who declared doing it less than (2%, *n* = 61) or equal to before (45%, *n* = 2). Although for the rest of the behaviors, the patients rated their frequency as predominantly equal to before, the proportion of those who rated their behavior as more frequent than before was particularly high for spending time on their mobile phone (45%, *n* = 72), on the Internet (30%, *n* = 41) and smoking cigarettes (25%, *n* = 33). The highest percentage of patients who declared a behavioral pattern as less frequent than before the lockdown was with regard to alcohol use (23%, *n* = 31).

### 3.2. Relationships between Gambling during the Lockdown, Variables Related to the Pre-Lockdown Period and Life Conditions in the Lockdown

To analyze which factors could be associated with gambling frequency and problem gambling symptoms during the lockdown, we investigated the relationships with all the other variables in relation to the pre-lockdown period and assessed during the lockdown. Only significant relationships are reported in Table 5. Both gambling frequency and problem gambling symptoms resulted in being negatively correlated with abstinence during the treatment, while only gambling frequency was negatively correlated with relapses. The positive perception of familial relationships during the lockdown was negatively related to problem gambling symptoms, while depressive feelings were positively related to problem gambling symptoms. The number of known persons who died because of the COVID-19 disease was positively related both to gambling frequency and problem gambling symptoms. Problem gambling symptoms were also negatively related to the frequency of alcohol use and positively correlated with the use of videogames. More frequent gambling behavior and higher gambling problem severity were associated with higher gambling craving. Moreover, gambling frequency and problem gambling symptoms were significantly and positively correlated (*r* = 0.38, *p* < 0.001).

### 3.3. Analysis of Problem Gambling Symptoms Change from the Pre-Lockdown Period to the Lockdown

Finally, in order to evaluate the change in problem gambling symptoms from the period before the quarantine to the lockdown, through a paired sample *t*-test, we compared the SOGS total score before and during the lockdown for the subjects who had the SOGS evaluation before the quarantine. We obtained a significant difference (*t*(42) = 16.42, *p* < 0.001) associated with a high effect size (Cohen’s *d* = 2.51). In particular, we observed a decrease in the mean score from pre-quarantine (M = 10.51, SD = 3.13) to during the lockdown (M = 1.23, SD = 1.32).

Starting from that premise, for each of those participants, we computed a delta score, indicating the difference across time in the SOGS total score. Thus, positive values would indicate a decrease in the total score from the period before the quarantine to the lockdown, and higher values would indicate greater change. The analyses showed that only one patient increased the SOGS total score (ΔSOGS = −3), while all the others had positive ΔSOGS values; thus, there had been a decrease in problem gambling symptoms. In particular, the mean value of ΔSOGS was 9.29 (SD = 3.70, range: −3–17). We also observed that this delta value was significantly correlated to the total years under treatment (*r* = 0.43, *p* < 0.01), having open spaces in the house (*r* = 0.37, *p* < 0.05) and the number of reasons for exiting the home (*r* = −0.47, *p* < 0.001). Moreover, it was negatively related to gambling craving (*r* = −0.36, *p* < 0.05). Thus, a more robust treatment pathway and a domestic environment that favors open spaces were associated with a higher decrease in problem gambling symptoms, while going outside for a lot of reasons seems not to favor a change. However, decreasing the problems related to gambling was related to a decrease in gambling craving.

## 4. Discussion

The pandemic has provoked a distinctive scenario and has generated new and unpredictable conditions for the entire population in general and for PGs in particular. During the quarantine, almost all land-based gambling locations were closed, making it impossible to go to places where people normally gambled before the lockdown. Taking into account this specific scenario in Italy, this study presents the results of in-depth telephone interviews that were administered to a sample of PGs under treatment by their referring healthcare professionals in order to assess their functioning during the rigid quarantine period caused by the COVID-19 pandemic. As far as we know, no other similar studies have yet been carried out in Italy or abroad.

The focus of our study is a population of PGs in the intermediate to advanced phase of their outpatient treatment. The majority of the patients interviewed were abstinent at the time of the start of the lockdown and are sufficiently representative of the patients being treated by the Italian addiction services. Overall, our patients were living in good conditions and the majority reported a general positive emotional state and good family relations. They lived mostly in medium-size houses with open spaces and with other people; most of them were still working during the lockdown, even on-site, and could continue to move outside their home using their money. Even though none of our sample became sick because of COVID-19, almost half of the patients knew someone that was affected, and one in ten had been faced with the deaths of several known people, caused by the virus. Fear, stress and anxiety were strongly elicited by the pandemic, and most of the patients expressed positive attitudes towards the government’s restrictions.

Interestingly, the vast majority of patients remained abstinent from gambling during the lockdown, despite the fact that going out for shopping and to work remained unchanged for most of the respondents, and they did not shift from land-based to online gambling. This result is particularly challenging considering that during the lockdown, several online gambling companies around the globe announced record numbers of people signing up and gambling. Various explanations of this fact are possible, depending on whether we focus on a clinical sample of problem gamblers being treated at services such as ours or on the overall population of gambler customers of online platforms. In fact, our patients were mostly land-based EGM-disordered gamblers. In line with Avanzi and colleagues [52], their preferred game may not have been interchangeable for them, they may have low skills with technology and, at the same time, family control may be greater during this period. Moreover, they could benefit not to be triggered by online gambling advertisements, forbidden in Italy since 2019 [53]. Instead, concerning the general population, data on online gambling released in Italy refers to an increase in cash revenue without knowing whether it is determined by the same gamblers who invested more money during lockdown than before or if there was an increase in the number of customers, or both. Regardless of the lockdown, a general constantly growing trend of online gambling revenue, notably +35% in the last three years, had already been registered in the last few years in Italy [54].

Only a modest switch towards other potential addictive behaviors, mainly to occupy the time during the compulsory stay at home (TV, use of smartphones and Internet and tobacco smoking), was found. The most relevant result confirms our hypothesis. The forced abstinence from gambling, imposed by the lockdown and the closure of most of the land-based gambling opportunities, has allowed many PGs in treatment to achieve a significant improvement in their quality of life rather than leading them to withdrawal syndrome or other adverse symptoms that might also have been expected. In fact, almost no PG symptoms were found, with the exception of the awareness of having a gambling problem and the sense of guilt because of gambling. We also observed low craving, a very limited shift towards other addictive behaviors to deal with the situation, improvement in relations with others and new plans for the future.

There has been a substantial culling of regular gambling since the pre-COVID-19 quarantine period (hardly anyone gambles any more). During the quarantine, patients had far fewer symptoms according to the diagnosis of GD, averaging below the diagnostic threshold and with a very significant problematic criteria reduction compared to the beginning of treatment. The longer the treatment, the more monitoring and accompaniment are present and the better the results in terms of symptom reduction from before to after the COVID-19 lockdown period. Some individual characteristics (years of taking charge), individual attitudes and behaviors related to quarantine (perception of government regulations and number of exits) and environmental characteristics (having open spaces) favor the reduction in symptoms. Associated with symptom reduction, the level of craving in our study resulted in being low overall.

In the field of addictions, strong support for the link between craving and substance use has been found—it is considered one of the key symptoms in addicted patients, closely correlated with the prognosis and progression of the pathology. Furthermore, lower levels can positively influence the treatment outcome, and craving is a known predictor of relapse after treatment [55]. Martinotti and colleagues [56], assessing an Italian sample affected by substance use disorder during the COVID-19 lockdown, also found low craving and explained it by “a perceived lack of availability of the substance that interrupted the development of the craving priming as well as decreased social pressure on this group of subjects” [56] (p. 6). We can assume the same perspective—the lack of availability of gambling opportunities—hindered the development of craving in the PG patients interviewed in our study, in a generally decreased social pressure environment.

In terms of craving intensity, the benefits of the presence of strict limitations on personal freedom, including the impediment to gambling, combined with the benefits of having had intensive treatment in addiction services, are an interesting result of our study that has relevant public policy implications, suggesting the need for necessary preventive environmental protective measures to support pathological gamblers’ treatments. Gainsbury and colleagues [57] found that compared to the other groups, among land-based gamblers, there was a higher proportion of PGs than in online gamblers, and land-based gamblers were also most likely to play electronic gaming machines weekly, with this form of gambling contributing to problems at a substantially greater rate. Restrictions on the general availability of land-based gambling, on who can gamble and on how gambling is provided are indeed best practices to prevent problem gambling [14], and our study shows that PGs might be effectively supported in their recovery if they are restricted and not allowed to gamble. St-Pierre and colleagues [58], in their review about the influence of availability and accessibility of gambling opportunities on problem gambling, noted that even though there is no robust evidence that increased availability and accessibility of gambling contribute to the prevalence of gambling pathology and problems, there is also no convincing evidence to the contrary. Indeed, our study highlights that decreased availability led to decreased problems.

In our study, we also found an intense feeling of relief due to the impossibility of gambling (i.e., the zeroing of access seems to be very effective in producing containment and well-being). This is an unexpected result of our study: having no gambling opportunities available is experienced mainly with a sense of freedom rather than being perceived as a limitation, which gives space to new interests, goals and relationships and family roles. Indeed, a significant number of gamblers reported a general mood of overall well-being. Social relationships in the family improved for half of the patients. The quality of life with cohabitants is, on average, evaluated as positive. These results differed from those found regarding alcohol. For instance, Chodkiewicz and colleagues [59], in their survey of alcohol-drinking throughout the pandemic in Poland, found that current alcohol drinkers were significantly less able to find anything positive about the pandemic situation and were mentally less able to cope. Those drinking more during lockdown were already heavy drinkers and suffered from worse mental health. They also used substances to cope with stress. Their levels of mental health were also reduced, especially in daily functioning and symptoms of depression. Similarly, Sun and colleagues [60] made the assumption that the COVID-19 pandemic has increased the risk of the abuse of addictive substances and behaviors. They found that relapses were relatively common in ex-alcohol (19%) and ex-smoking addicts (25%); furthermore, 32% of regular alcohol drinkers and 20% of regular smokers increased their usage amount during the pandemic. These three dysfunctional coping behaviors (Internet, alcohol and smoking) during this COVID-19-related crisis appeared to have increased the risk for substance use disorders and Internet addiction in previous consumers.

A slight shift towards other potential addictive behaviors to cope with the situation has been observed in our study, but this should be considered quite normal in a period of quarantine as dysfunctional coping strategies to face it were found also in the general population [61,62,63,64,65,66]. It is also important to note that public stress, anxiety, grief, isolation and financial problems could have a large role in the proliferation and ongoing relapse of substance misuse and behavioral addiction [60,67].

## 5. Implications for Treatment

The great majority of the respondents have not gambled, nor has their symptomatology worsened; therefore, not finding gambling offers seems to support their treatment. Of extreme clinical interest are the perspectives that the interviewees expressed about their future intentions and about the lack of attention that they have experienced with respect to gambling. The images of what they would have liked to do at the end of the quarantine focus on desires common to people who have never experienced gambling addiction (e.g., seeing friends, playing sports, taking vacations, finding or starting work, etc.) but which are not common for PGs who usually have the overriding compulsion to gamble, which annihilates all other desires and projects. This is consistent with the many respondents who have lost interest (11%) or felt they hate gambling (8%) during lockdown. Therefore, the closure and consequent inaccessibility of most land-based gambling opportunities has allowed these patients to return to desire and plan for their future life outside the arcades, away from gambling, reversing that process of polarization of thought and of the whole life focused on gambling, which is the first indicator of loss of control and sliding into GD [68]. Some patients also verbalized the desire to maintain these changes in life.

However, a few people have experienced increased craving and a few gambling episodes. Again, this can shed light on the process of relapse, from both a preventive and a clinical perspective. In our clinical sample, relapses during the lockdown period are associated mainly with an inhomogeneous treatment pace and with the traumatic experiences associated with COVID-19. In this context, we have observed that stress and negative emotions act as triggers, but mostly in patients who had unstable gambling abstinence. Most interestingly, the small subgroup that had a relapse was composed only of patients who were habitual gamblers of those options that were still available during the lockdown (scratch cards, online gambling and the stock market) and had grieving experiences; on the other hand, there was no shift to other forms of gambling for all the other patients. Thus, environmental influence plays an important role in relapse and has a crucial role in potentiating the effects of the treatment and allowing different forms of coping with stressful situations.

Some interesting findings that emerged from the study also highlight some general difficulties experienced by gambling patients during quarantine. Some of what Browne and colleagues [69] call “legacy harms” were also found (i.e., harm to the individual’s emotional state, ongoing guilt and shame and ongoing financial harm). One in ten had deteriorated social or family relationships. This could be explained by the fact that in some households, there are additional issues that aggravate the conflict between members (e.g., tensions and domestic violence, but also economic problems that carry over from the period of heavy gambling). In fact, economic problems may also have worsened during the quarantine period, despite the suspension of gambling behavior: economic concerns, in addition to the decrease in work and the economic crisis due to the pandemic, may be linked to the fact that the gambler can no longer count on the income from before and therefore may not be able to pay previously open debts that continue to arrive (for example, the payment of loan instalments previously requested from financial companies). This can result in feelings of malaise, economic worries and anxiety. Symptoms of ongoing disordered gambling awareness and guilt also persist, regardless of not having gambled during the quarantine period. The sense of guilt, disappointment, anger and remorse for the lost money remains high; the other dominant feeling is indifference to gambling. In line with what Volkow said [66], it is therefore necessary to continue supporting and monitoring addicted gamblers in recovery and their concerned significant others, compensating the limited access to meetings of peer-support groups or other sources of social connection with other forms of support. Although face-to-face interaction is a key feature of recovery support, virtual meetings may be useful for those with access to the Internet, and enhancing virtual resources to minimize office visits appeared to be an effective strategy for supporting patients during the quarantine period.

Finally, many interviewees are aware that imminent exposure to post-quarantine gambling will put them back in a risky situation, and a dozen respondents expect that they will return to their previous gambling habits. As Ben Cave Associates and the Southwark Council [70] report, there is a reasonable body of scientific evidence that shows links between access to gambling venues, betting shops and gambling locations and increased gambling and problem gambling behavior, which also leads to poor health outcomes. Therefore, features of the environment such as location and number of gambling opportunities in a specified area are recognized as variables that often facilitate and encourage people to gamble, important in both the initial decision to gamble and in the maintenance of the behavior.

## 6. Conclusions

Forced abstinence from gambling, together with the presence of family members and with an international climate of health emergency, seems to improve the quality of life of PGs under treatment and even favors intentional responsibility towards the future, which, however, may not be sufficient in itself to maintain the abstention from gambling. Even if individual tools (resilience, self-efficacy and self-esteem) can facilitate this intention, especially when combined with long-lasting treatment paths, environmental tools (i.e., protection from gambling places) seem to be particularly important to maintain this intention, to prevent relapses and to achieve a better quality of life and well-being, despite extreme, stressful and adverse situations, such as the pandemic.

Our data are in line with other studies that have monitored the results of the effects of restrictions on the gambling venues; for example, the Regional Law of Piedmont [71], which significantly limited the placement and number of EGMs with rules on zoning and timing, within a couple of years from its entry into force (2016–2018) achieved a drop in consumption and expenditure of 9.7% compared to an increase of 1.6% in the rest of Italy [72], a trend already noted in other European studies [73,74]. In line with Young and colleagues [75], therefore, there is now evidence to justify the explicit incorporation of geographic accessibility as a parameter to control the spatial distribution of gambling supply as a harm-reduction strategy.

This study has some limitations: our sample is not representative of all PGs—because the low rate of access to services is known, disordered gamblers in treatment are only a fraction of the total; several studies conducted around the world [76,77,78,79,80] agree that only between 7.1% and 29% of PGs seek formal help. Nevertheless, our sample is wide and representative of the PGs who turn to addiction services for help. Moreover, it was possible to compare the SOGS scores before and during quarantine for only 44 patients, since the measuring instruments administered to the intake by the various addiction services differed, and because the adherence to the study by the services occurred when the lockdown had already begun. Therefore, it was not possible to agree on univocal measures pre- and post-enrolment of the patients in the research design. Nevertheless, all patients had received a diagnosis of GD according to DSM criteria. For the same reason, we did not have a measure of severity of the immediate period before the lockdown. Finally, it was not possible to compare our data with a control sample, given the simultaneous closure of almost all gambling opportunities in Italy.

Long-term studies with follow-up at the end of the restrictive measures due to the pandemic and by its socio-economic consequences may clarify the true impact of the COVID-19 pandemic on those subjects affected by GD, and associated systematic research studies with longer observation periods and control groups, wherever possible, are essential. Further research monitoring the support given by the health services for both patients and family members are also appropriate to reach this goal.

This study opens up interesting questions in terms of environmental prevention and structural harm reduction. Since 2019, an Italian law [53] prohibits commercial advertising for gambling, and we assume that this may probably have facilitated the reduction in craving of gamblers. Therefore, it is necessary to at least provide forms of voluntary self-exclusion and imposed exclusion for pathological land-based gamblers, according to the evidence from the numerous efficacy studies carried out in casinos, venues and online betting sites and virtual casinos in terms of harm reduction for this vulnerable population [81,82,83,84,85]. This study suggests preventive effects of substantial reductions in supply in the field of games of chance. Consequently, a substantial reduction in the number of gambling opportunities in Italy should be considered as justified from a research perspective: public policy should therefore consider every form of quantitative restriction or strict limitation on the supply of games of chance as a component of the regulation of gambling for the purpose of protecting the gamblers, especially those belonging to vulnerable categories.

## Figures and Tables

**Table 1:** Anamnestic information of the gamblers prior to the lockdown.

Variables				
Years of taking charge	M		SD	Range
	6.96		6.43	0–15
Treatment phase	Acceptance			9% (*n* = 12)
	Clinical treatment			41% (*n* = 55)
	Monitoring			38% (*n* = 52)
	Pre-discharge			11% (*n* = 15)
	Discharge			1% (*n* = 1)
Treatment approach	Psychological (individual/in group)			78% (*n* = 105)
	Psychological + Pharmacological			10% (*n* = 13)
	Psychological + Psychoeducational			4% (*n* = 5)
	Psychoeducational + Economic tutoring			7% (*n* = 10)
	Psychological + Pharmacological + Psychoeducational + Economic tutoring			1% (*n* = 2)
Abstinence (at the time of taking charge)	No		33% (*n* = 45)	
	Yes		67% (*n* = 90)	
Abstinence (during treatment)	No		26% (*n* = 35)	
	Yes		74% (*n* = 98)	
Relapses	No		50% (*n* = 68)	
	Yes		50% (*n* = 67)	
Involvement of family in the treatment	No		31% (*n* = 42)	
	Yes		69% (*n* = 93)	
Dominant gambling activity	Slot machines/other gambling machines		78% (*n* = 106)	
	Instant scratch-cards		8% (*n* = 11)	
	Stock and/or commodities market bets		7% (*n* = 9)	
	Sport bets		1% (*n* = 1)	
	Traditional lotteries		3% (*n* = 4)	
	Poker for money		1% (*n* = 1)	
	Online gambling		2% (*n* = 3)	
Gambling frequency	Non-regular		79% (*n* = 104)	
	Regular		8% (*n* = 10)	
	No gambling behavior		13% (*n* = 11)	
Number of gambling activities	M		SD	Range
	1.85		1.19	1–10
Pathway	Pathway 1		41% (*n* = 51)	
	Pathway 2		52% (*n* = 65)	
	Pathway 3		7% (*n* = 8)	
Comorbidities	No		57% (*n* = 74)	
	Yes		43% (*n* = 56)	
	M	SD	Range	
	1.12	0.80	1–2	
	Depressive disorders		36% (*n* = 20)	
	Personality disorders		18% (*n* = 10)	
	Anxiety disorders		12% (*n* = 7)	
	Bipolar disorder		7% (*n* = 4)	
	Substance-related disorders		9% (*n* = 5)	
	Neurocognitive disorders		2% (*n* = 1)	
	Psychosis/Schizophrenia		12% (*n* = 7)	
	Obsessive-compulsive disorder		2% (*n* = 1)	
	Eating disorder		2% (*n* = 1)	

**Table 2:** COVID-19 disease personal contacts and perception.

Variables					
Personal disease	No			100% (*n* =135)	
	Yes			-	
Quarantine	No			96% (*n* = 130)	
	Yes			4% (*n* = 5)	
Knowledge of affected persons	No			59% (*n* = 79)	
	Yes			41% (*n* = 56)	
Number of affected persons	M	SD		Range	
	3.50	6.82		1–50	
Knowledge of dead persons	No			90% (*n* = 121)	
	Yes			10% (*n* = 14)	
Number of dead persons	M	SD		Range	
	3.14	4.24		1–15	
Emotional impact	Emotions		M	SD	Range
	Anxiety		4.39	3.06	1–10
	Depression		3.05	2.89	1–10
	Fear		4.69	3.13	1–10
	Stress		4.58	3.20	1–10
	Anger		4.14	3.28	1–10
	M	SD		Range	
Eating problems	2.49	2.97		1–10	
Sleeping problems	3.68	3.16		1–10	
Attitude towards the government’s restrictions	M	SD		Range	
	27.17	4.38		15–35	
Exits from home in the last week	Never			4% (*n* = 6)	
	1–3 times			32% (*n* = 43)	
	4–6 times			28% (*n* = 38)	
	7–9 times			26% (*n* = 35)	
	More than 10 times			10% (*n* = 13)	
Reasons to exit from home in the last week	Walking alone			18% (*n* = 24)	
	Walking with someone of my family			2% (*n* = 3)	
	Walking with friends			-	
	Returning to the own residence			2% (*n* = 2)	
	Going to work			44% (*n* = 59)	
	Doing the shopping			73% (*n* = 99)	
	Running urgent errands			7% (*n* = 9)	
	Going to the pharmacy			19% (*n* = 26)	
	Going to the hospital			2% (*n* = 3)	
	Going to the doctor			3% (*n* = 1)	
	Assisting family/friends in need			8% (*n* = 11)	
Total number of reasons to exit from home in the last week	M	SD		Range	
	1.96	1.09		0–5	

**Table 3:** Endorsement by problem gambling symptoms of the South Oaks Gambling Screen (SOGS).

Symptom	f% (f)
Going back another day to win money back	2% (*n* = 2)
Claiming to be winning money gambling but it was not real	2% (*n* = 3)
Feeling to have a gambling problem	84% (*n* = 106)
Gambling more than intended to	2% (*n* = 3)
Having people who criticize own gambling	7% (*n* = 9)
Feeling guilty because of gambling	10% (*n* = 13)
Feeling like one would like to stop gambling but he/she could not	4% (*n* = 5)
Hiding betting slips	4% (*n* = 4)
Having money arguments centered on gambling	5% (*n* = 7)
Borrowing money to gamble and not paid them back	5% (*n* = 6)
Losing time from study/work to gamble	1% (*n* = 1)
Borrowing money to gamble	2% (*n* = 2)

**Table 4:** Emotional state towards gambling.

“How Do You Feel about Gambling during this Period of Lockdown?”	f% (f)
Indifference (“I think about other things”)	32% (*n* = 44)
Guilt (“I feel disappointed for the money I lost gambling”)	24% (*n* = 32)
Freedom (“I feel free from gambling”)	11% (*n* = 15)
Loss of interest (“I put gambling aside”)	11% (*n* = 15)
Disgust (“I hate gambling”)	8% (*n* = 11)
Anger (“I am very angry when thinking about gamblingI am very angry thinking to gambling”)	6% (*n* = 8)
Fear (“to I am worried thinking about when gambling venues will be opened I am worried thinking to when the gambling venues will be open”)	5% (*n* = 7)
Ambivalence (“I feel disgust but also desire”)	1% (*n* = 1)
Protest (“I am very angry at the locking of gambling venues I am very angry for the lock of gambling venues”)	1% (*n* = 1)
Boredom (“I’m bored without gambling”)	1% (*n* = 1)

**Table 5:** Significant correlations between gambling frequency and problem gambling during the lockdown, with variables related to the pre-lockdown period and the patients’ life conditions during the lockdown.

Variables	Gambling Frequency	Problem Gambling Symptoms
Abstinence during treatment	*r* = −0.28 ** (*n* = 133)	*r* = −0.21 * (*n* = 126)
Relapses	*r* = 0.24 ** (*n* = 135)	*r* = 0.14 (*n* = 127)
Number of known persons died for COVID-19	*r* = 0.18 * (*n* = 135)	*r* = 0.24 * (*n* = 127)
Positive perceptions of familiar relationships during the lockdown	*r* = −0.07 (*n* = 103)	*r* = −0.30 ** (*n* = 99)
Depressive feelings	*r* = 0.06 (*n* = 115)	*r* = 0.21 * (*n* = 110)
Alcohol use frequency	*r* = 0.02 (*n* = 131)	*r* = −0.22 * (*n* = 127)
Videogames use frequency	*r* = −0.04 (*n* = 135)	*r* = 0.24 ** (*n* = 127)
Gambling craving	*r* = 0.21 * (*n* = 127)	*r* = 0.37 * (*n* = 127)

* p < 0.05, ** p < 0.01.

## Data Availability

The data presented in this study are available on request from the corresponding author.

## Supplementary Materials

The following are available online at https://www.mdpi.com/1660-4601/18/2/424/s1, Table S1: Questions and response options of the telephone Interview.
