# Supplementary material for: Being a Gambler during the COVID-19 Pandemic: A Study with Italian Patients and the Effects of Reduced Exposition
Source: Int J Environ Res Public Health. 2021 Jan 7;18(2):424. doi: 10.3390/ijerph18020424 (PMC7825745; doi:10.3390/ijerph18020424)
Supplement: Supplementary file 1 [file ijerph-18-00424-s001.pdf]

SUPPLEMENTARY MATERIAL

Questions and response options of the telephone Interview

Section	Variable	Questions and response options
1	Home environment	"How many square meters is the house where you live?" [open question];<br><br>"Are there open spaces in the house?" [*yes*, *no*]; "If yes, which of the following? [*garden, terrace, vegetable garden*];<br><br>"How many people are you living with? [open question].
	Familiar relationships	"Please rate the relationships with your family members from a minimum of *1 – worst*, to a maximum of *10 – excellent*)".
	Job conditions	"Which of the following is your working condition at the moment?" [*job interruption, work on-site, smart working*].
	Money management	"During the last month, have you ever made use of money?" [*yes*, *no*]; "If yes, for what reasons?" [open question]; "With the supervision of family members?" [*never*, *sometimes*, *often*].
	Emotional state	"How did you feel during these last weeks?" [open question].
	Behavioral intentions for the post-quarantine	"What do you think you will do after the lockdown?" [open question].
2	Personal impact with the COVID-19 disease	"Have you got sick of COVID-19? [*yes*, *no*];<br><br>"Have you done the quarantine due to the contact tracing?" [*yes*, *no*];<br><br>"Do you know people affected by the COVID-19?" [*yes*, *no*];<br><br>"Do you know people who had died because of the COVID-19?" [*yes*, *no*].
	Perceived emotional impact of the COVID-19 pandemic	"Please rate how do you felt, from a minimum of *1* (*very little*) to a maximum of *10* (*very much*), regarding each of the following emotional states: anxiety; depression; fear; stress; and anger";<br><br>"Please rate how do you had eating and sleeping problems, from a minimum of *1* (*very little*) to a maximum of *10* (*very much*)".
	Perception of the adequacy of the national restrictions	"Please report how do you agree with the following statements using a 5-point Likert scale from 1 (*totally false*) to 5 (*totally true*)".
	Behavior adhered to the rules	"How frequently did you exit from home in the last week? [*none*, *1–3 times*, *4–6 times*, *7–9 times*, *more than 10 times]*;<br><br>"For which reasons among the following? [*walking alone*; *walking with someone in my family*; *walking with friends*; *returning to the own residence*; *going to work*; *doing the*

		shopping; *running urgent errands*; *going to the pharmacy; going to the hospital*; *going to the doctor*; *assisting relatives/friends in need].*
3	Gambling problem severity	SOGS
	Emotional states towards gambling	"*How do you feel about gambling during this period of lockdown?*" [open question].
	Gambling craving	"Please report how do you agree with the following statements using a 7-point Likert scale from *strongly disagree*) to 7 (*strongly agree*).
4	Other potentially addictive behaviors	"How frequently did you have the following behaviors during the last month?" [*never*, *sometimes*, *often].*
5	Perceptions about changes from the pre-lockdown period to the ongoing lockdown period	"By referring to this lockdown period, do you think the relations with the family members are?" [*improved*, *worsened*, *remain the same*]"; "Why?" [open question].<br><br>"With respect to the period prior to the COVID-19 pandemic, how do you feel? [*better*, *worse*, *the same*]"; "Why?" [open question].<br><br>"With respect to the period before the beginning of the lockdown, have you made more or less frequent use of money under the supervision of family members?" [*less than before*, *more than before*, *equal*];<br><br>"With respect to the period before the beginning of the lockdown, have you had more or less frequently the following behaviors? Alcohol use, videogame playing, Internet use, smoking, substance use, online shopping, TV watching, and smartphone use?" [*less than before*, *more than before*, *equal*].
